# Supplementary material for: CXCL8 Up-Regulated LSECtin through AKT Signal and Correlates with the Immune Microenvironment Modulation in Colon Cancer
Source: Cancers (Basel). 2022 Oct 28;14(21):5300. doi: 10.3390/cancers14215300 (PMC9657600; doi:10.3390/cancers14215300)
Supplement: Supplementary file 1 [file cancers-14-05300-s001.zip › cancers-1954068-supplementary.pdf]

CXCL8 Up-regulated LSECtin through AKT Signal and Correlates with the Immune Microenvironment Modulation in Colon Cancer

Running title

Role of CXCL8 on LSECtin and immune microenvironment

Authors

Shaojun Fang [1 †], Xianshuo Cheng[1 †], TaoShen[1], Jian Dong [1], Yunfeng Li[1], Zhenhui Li [2], Linghan Tian[4], Yangwei Zhang[1], Zhengfeng Yin [3], ZhibinYang[1*]

Original data for WB. Full-length original images of Western blots showing the effects of CXCL8 on AKT signaling proteins and LSECtin expression in SW480 and SW620 cells (in three groups: "PBS, CXCL8+MK2206, CXCL8" or "Wild type, Normal control, CXCL8 RNAi-2"). Blots surrounded by the red border were corresponding to the Figure 8 in the same order and locations. Blots not surrounded by the red border were other experiments. The expression of other proteins which had different molecular weight when conducting the WB ECL-HRP detection process should be detected for other researching purpose. Thus, to avoid acquiring multiple protein bands in one field, which would interfere the results presentation of this study, protein bands which considered to be interest protein were cut prior to hybridisation with antibodies according to the hint of protein marker.

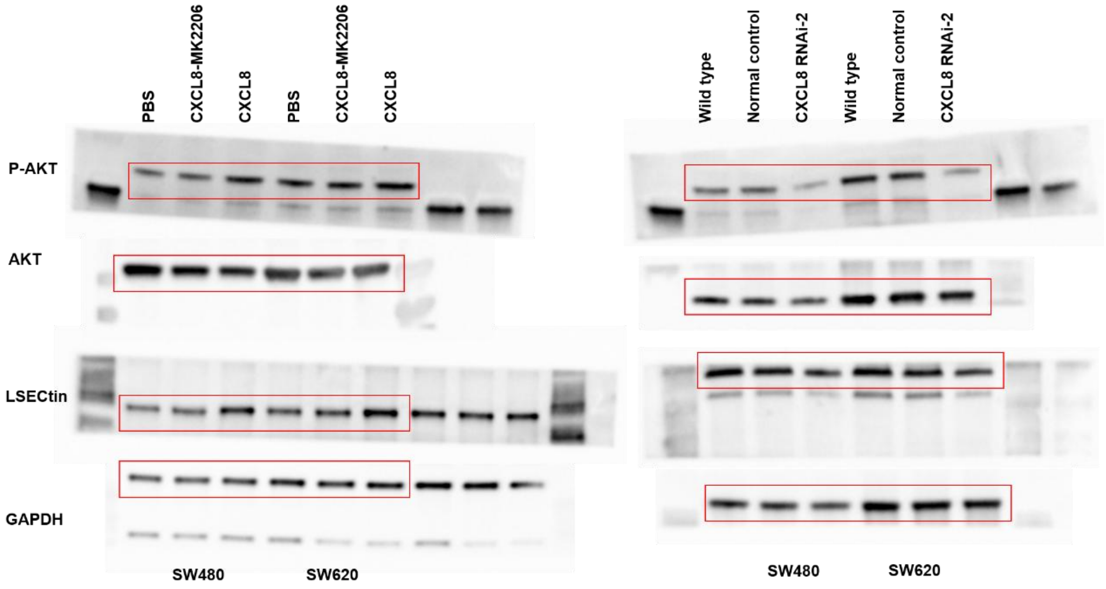

PBS
CXCL8-MK2206
CXCL8
PBS
CXCL8-MK2206
CXCL8
Wild type
Normal control
CXCL8 RNAi-2
Wild type
Normal control
CXCL8 RNAi-2
P-AKT
AKT
LSECtin
GAPDH
SW480
SW620
SW480
SW620
